# Selective feeding of three bivalve species on the phytoplankton community in a marine pond revealed by high-throughput sequencing

**DOI:** 10.1038/s41598-022-08832-7

**Published:** 2022-04-13

**Authors:** Ling Qiao, Zhiqiang Chang, Jian Li, Tiejun Li

**Affiliations:** 1 Key Laboratory of Sustainable Utilization of Technology Research for Fishery Resource of Zhejiang Province, Zhejiang Marine Fisheries Research Institute, Zhoushan, 316012 China; 2 Key Laboratory of Sustainable Development of Marine Fisheries, Ministry of Agriculture and Rural Affairs, Yellow Sea Fisheries Research Institute, Chinese Academy of Fishery Sciences, Qingdao, 266071 China

**Keywords:** Molecular ecology, Animal behaviour

## Abstract

The study of the selective feeding of bivalves is necessary in order to improve our understanding of bivalve growth and development, which helps to better define the roles of bivalves in their ecosystems. Little information is currently available on the feeding preferences of bivalves in natural waters, since all diets are provided as single or mixed algae in experiments. In this study, high-throughput sequencing of the 23S rRNA gene was performed to explore differences in the feeding selectivity of *Mercenaria mercenaria*, *Meretrix meretrix* and *Ruditapes philippinarum* during different stages of their culturing to reveal their feeding preferences in natural waters. We found that the three bivalve species had different preferential selection of phytoplankton genera, indicating specific selection and avoidance of particular types of algae during their development in aquaculture. *M. mercenaria* was the most selective of the bivalves, followed by *M. meretrix* and then *R. philippinarum*. With the growth of *M. mercenaria* and *M. meretrix*, more kinds of phytoplankton could be ingested. In addition, high-throughput sequencing showed that some picophytoplankton including *Synechococcus*, *Microchloropsis,* and *Chrysochromulina* were dominant in the hepatopancreas samples obtained from these three bivalves. Therefore, the importance of these pico-sized algae in bivalve diets should be reassessed.

## Introduction

Bivalves are the major species of mariculture in China^1^. In 2020, bivalve production was about 14.80 million tons, which accounted for approximately 69% of the total Chinese mariculture production^2^. They are remarkable not only for their contribution as a food source for people, but also for the significant environmental effects they have as water eutrophication reducers and carbon dioxide bio-sequestrators^3^. Through the uptake and assimilation of particulate organic matter and phytoplankton, bivalves accumulate nutrients in their tissues, which removes these compounds from the ecosystem when they are harvested. Hence, bivalves have been widely used to regulate and mitigate eutrophication and other undesirable environmental conditions^4–6^. Marine bivalves also play an important role in the carbon cycle because bivalves can sequester carbon in their shells, which can act as CO_2_ sinks^7^. A conservative extrapolation of individual bivalve budgets to the global production results in a sequestration of 6.3 × 10^5^ tons of CO_2_ per year^8^. On the other hand, the “bottom-up” nutrient control of bivalves^9^ could effectively accelerate phytoplankton turnover and primary production rates, which directly increases the net CO_2_ fixation via photosynthesis, thereby accelerating carbon assimilation into the biosphere^8^. Therefore, bivalves have been extensively studied for their economic value and ecological function.

Filtering bivalves are herbivorous and consume microalgae throughout their lifespan^10,11^. Microalgae have high nutrient value, containing specific nutrient components such as pigments, essential fatty acids, and vitamins^12^, which satisfy the requirement for certain essential nutrients in bivalves^13,14^. Previous studies have revealed that bivalves display selective feeding on phytoplankton^15^, preferentially ingesting phytoplankton of suitable sizes and nutrient content, while rejecting those of low-quality or undesirable size; thus, optimizing their energy intake^15–17^. In addition, studies have also indicated that bivalves can discriminate between different microalgal species^15^. Recent work reported that this selection was mediated by interactions between lectins found in the mucus covering bivalve feeding organs and carbohydrates found on microalgae cell surfaces^18,19^. Elucidating the feeding selectivity of bivalves could help us understand their ecological niche and the role bivalves play in aquatic ecosystems^20^. These results would also provide an ecological basis for the rational utilization of microalgae resources and improve the yield of filter-feeding bivalves.

In studies of particle capture and selection in bivalves, differences in applied methodologies and experimental designs could be responsible for the differences in the reported research results^15^. In particular, the use of natural seston assemblages in field studies could lead to differences between results obtained using mixed microalgal diets in the laboratory^21,22^. Extensive laboratory feeding experiments of bivalves have been conducted and reported^17,23–25^. However, understanding the feeding selectivity of bivalves on phytoplankton in situ is challenging due to shortcomings of current detection methods. Traditionally, species identification from gut content analysis has been conducted using morphological methods. However, this is time consuming and identifying fragile, small and low abundance phytoplankton is difficult^26,27^. With the development of next-generation sequencing technologies in recent years, millions of amplified DNA fragments can be sequenced simultaneously, which provides a greater capacity to capture low-abundance DNA sequences^28^. Therefore, high throughput sequencing technology can give a reliable picture of the composition of phytoplankton ingested by bivalves, especially fragile phytoplankton that is otherwise easily deformed or destroyed during digestion, as well as pico-sized phytoplankton that cannot be identified by microscopy^29–31^. 18S rDNA is one of the commonly used marker genes for the study of feeding habits of aquatic animals^32–34^. The challenge encountered when using 18S rDNA to identify prey species is that predator DNA is frequently amplified along with prey DNA, which leads to a dilution of the desired prey sequence data^35^. The universal plastid amplicon (UPA) is a fragment of the plastid 23S rRNA gene and can be amplified from both eukaryotic algae and cyanobacteria^36,37^, but not bivalves in situ diet studies. Therefore, high-throughput sequencing technology using 23S rRNA provides an efficient tool for robust prey species identification of the diet of bivalves.

In this study, high-throughput sequencing based on 23S rRNA was performed to investigate the phytoplankton community in the water column and hepatopancreases of *Mercenaria mercenaria*, *Meretrix meretrix,* and *Ruditapes philippinarum* grown in aquaculture ponds. The objectives of the present study were to explore the differences in the feeding selectivity of three bivalve species during the different stages of culture, and to reveal the feeding preferences of different bivalves in natural waters.

## Results

### Water quality parameters and the weight variation of bivalves

**Figure 1:**
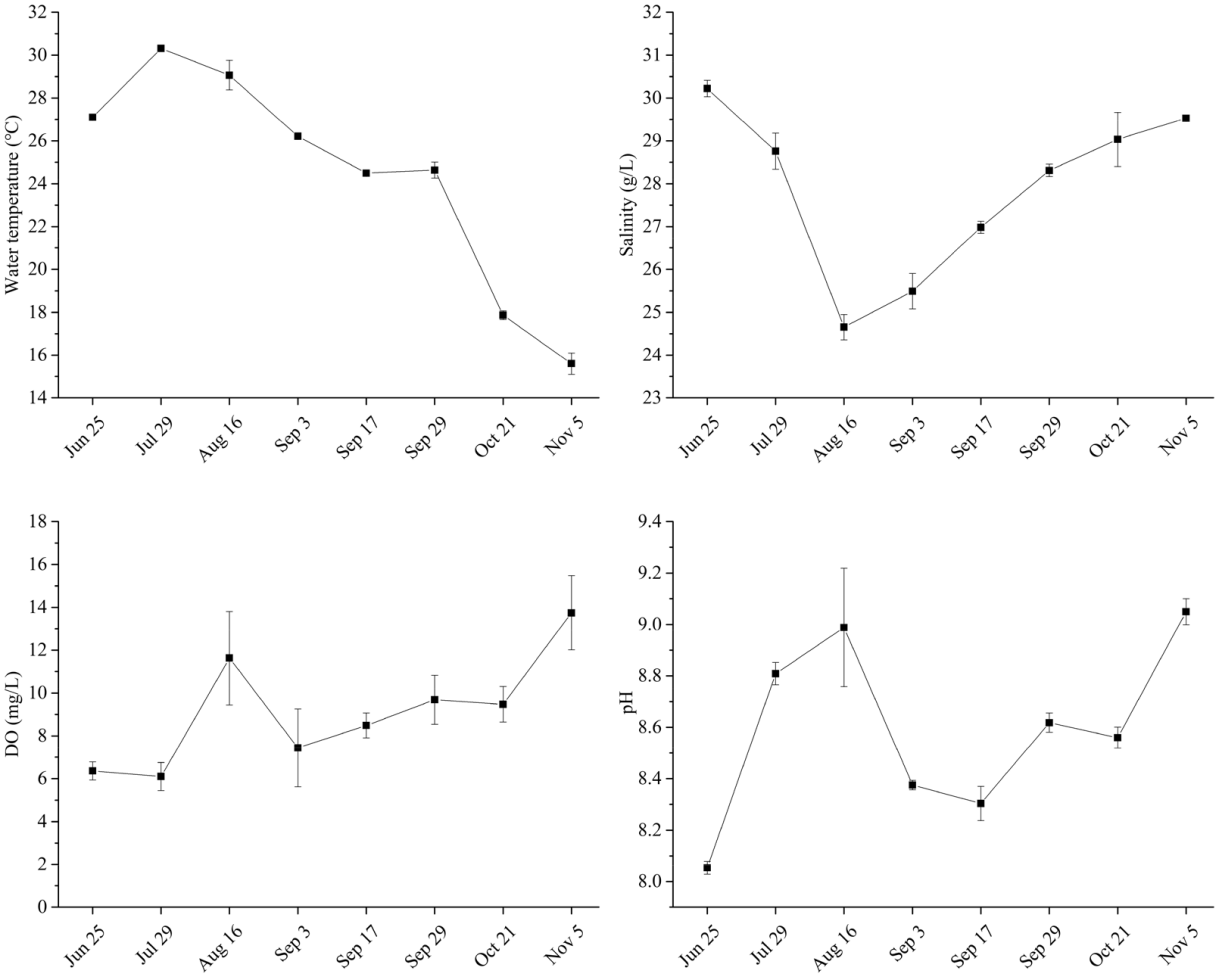
Water quality parameters in the aquaculture pond.

**Figure 2:**
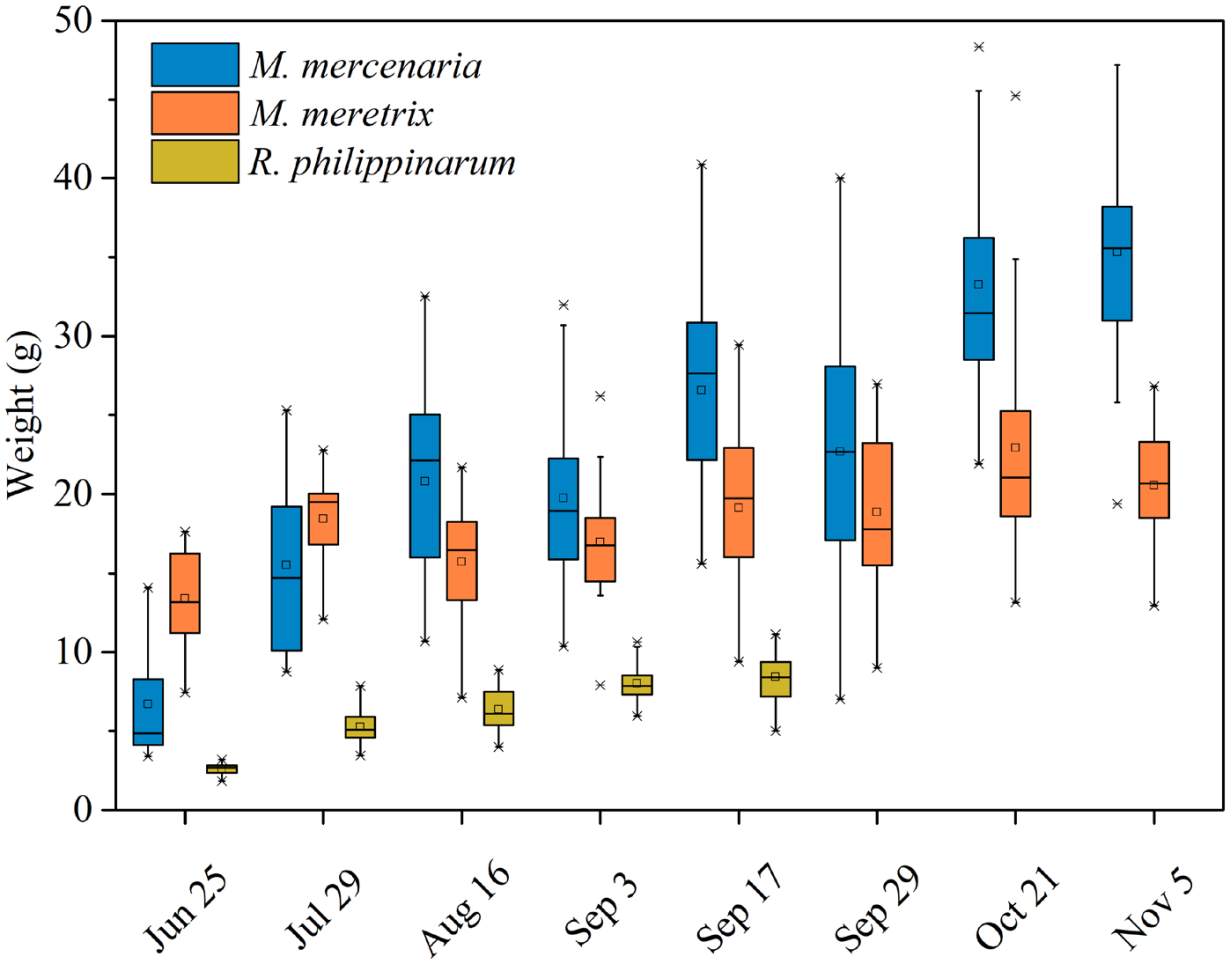
The weight variation of *M. mercenaria*, *M. meretrix*, and *R. philippinarum.*

The water quality parameters in the aquaculture pond are summarized in Fig. 1. During the study period, the water temperature increased firstly and then decreased, with maximum on 29 July (30.32 °C) and minimum on 5 November (15.60 °C). The salinity ranged from 24.65 to 30.22 g l^-1^, showing decreased firstly and then increased, with minimum value on 16 August. DO concentrations showed a trend of gradual increase, ranged from 6.10 to 13.75 mg l^-1^. The pH values ranged from 8.05 to 9.05, appearing increased firstly and then decreased and then increased again. During the study period, the average weight of *M. mercenaria*, *M. meretrix*, and *R. philippinarum* increased from 6.70 to 35.33 g, 13.41 to 20.53 g and 2.61 to 8.43 g, respectively (Fig. 2). At the middle and later stages of aquaculture, the average weight of *M. mercenaria* was highest, followed by *M. meretrix* and *R. philippinarum* (Fig. 2).

### Morphological analysis of phytoplankton abundance in aquaculture water

**Figure 3:**
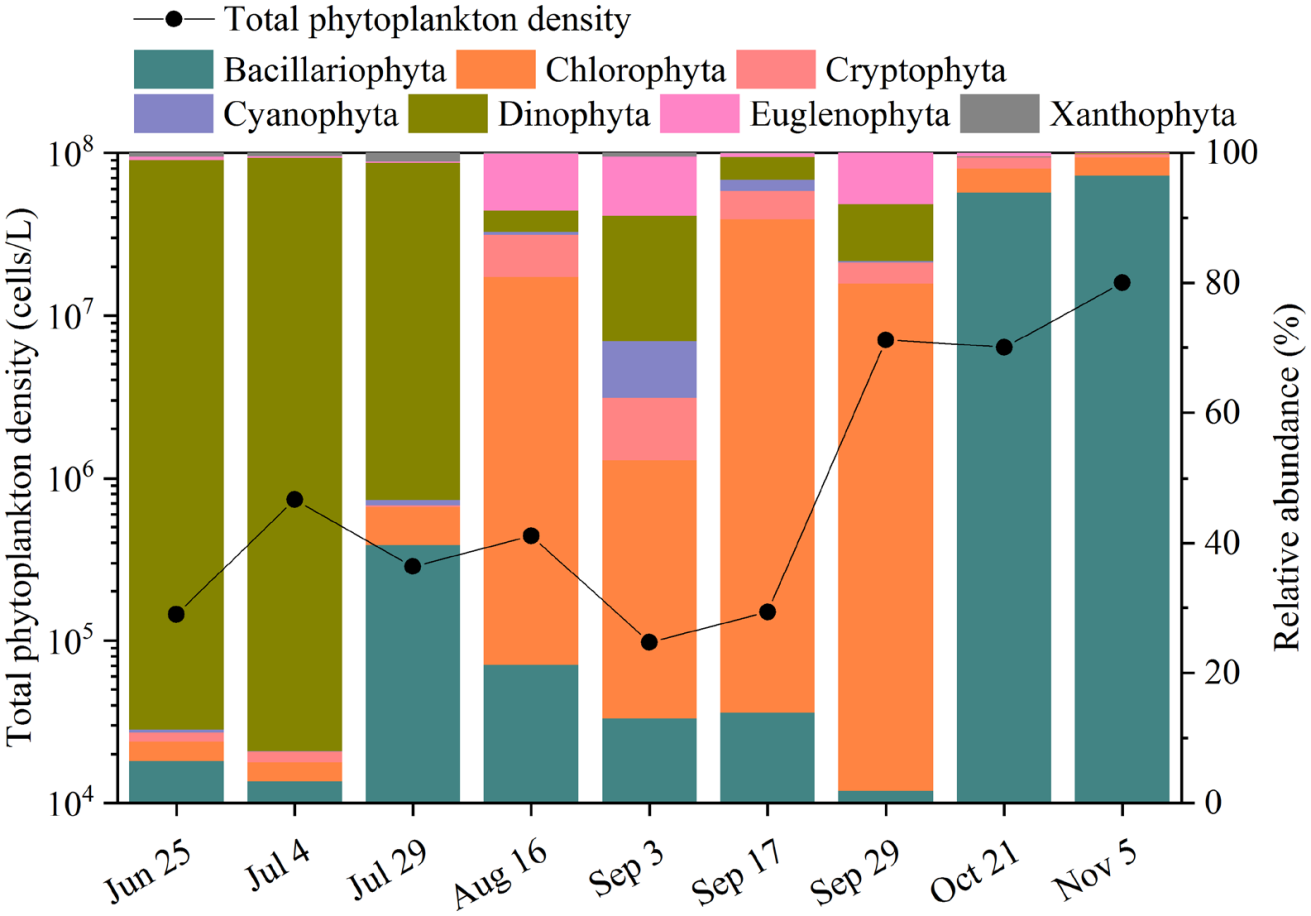
Morphological analysis of the temporal variation in total phytoplankton density and relative abundance in bivalve aquaculture water samples.

Phytoplankton abundance in the aquaculture water decreased and then increased from June to November, with a minimum of 9.76 × 10^4^ cells l^-1^ on 3 September and a maximum of 1.59 × 10^7^ cells l^-1^ on 5 November (Fig. 3). A total of seven phytoplankton phyla were identified in the aquaculture water by morphological analysis during the study period (Fig. 3). Dinophyta was the most abundant phylum, which contributed more than 51% of the total phytoplankton abundance from June to July. In August and September, the relative abundance of Dinophyta declined, while the relative abundance of Chlorophyta increased to 39.71–78.01% of the total phytoplankton abundance. From October to November, Bacillariophyta was the most dominant, accounting for more than 93% of the total phytoplankton community.

### High-throughput sequencing for the identification of phytoplankton community composition

**Figure 4:**
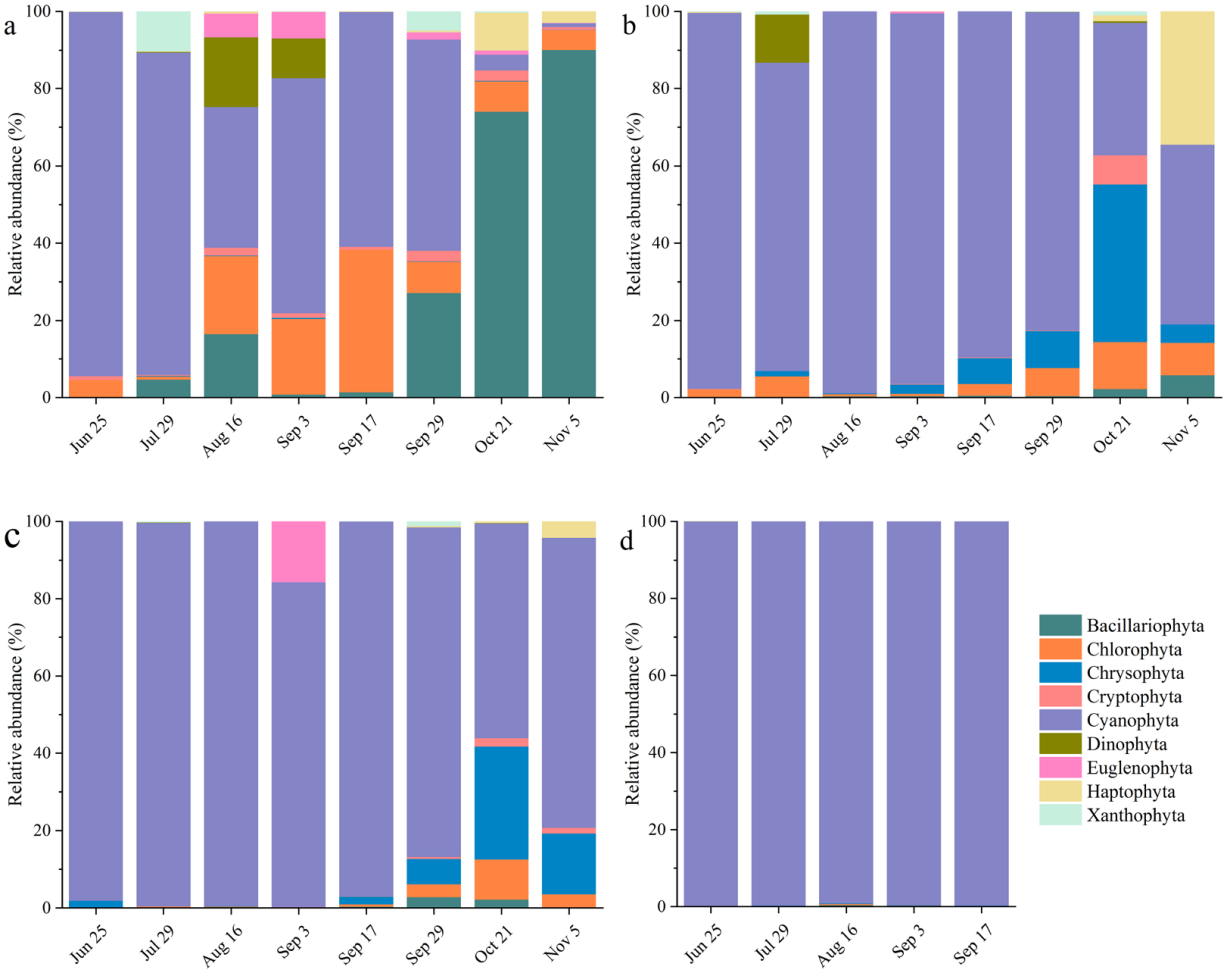
Temporal variation in the relative abundance of phytoplankton at the phylum level in aquaculture water samples (**a**), and hepatopancreases from *M. mercenaria* (**b**), *M. meretrix* (**c**), and *R. philippinarum* (**d**) revealed by high-throughput sequencing.

At the phylum level, a total of nine phyla were identified based on high-throughput sequencing (Fig. 4). In aquaculture water, Cyanophyta were the most dominant phylum, accounting for over 83% of the total phytoplankton from June to July. With the extension of aquaculture, the relative abundance of Cyanophyta declined (36.47%–60.90%) as the relative abundance of Chlorophyta increased (19.64%–36.91%). From late September to early November, the relative abundance of Bacillariophyta increased gradually, with a maximum of 90% on November 5 (Fig. 4a). In the hepatopancreas samples collected from *Mercenaria mercenaria* and *Meretrix meretrix*, Cyanophyta was the most dominant phylum (34.29%–98.95% and 55.56%–99.55%), followed by Chrysophyta (0.00%–40.79% and 0.00%–29.21%) and Chlorophyta (0.56%–12.26% and 0.00%–10.38%) (Fig. 4b, c). In the early and middle periods of the experiment, the relative abundance of Cyanophyta was the highest, with a maximum of 98.95% and 99.55% on August 16, respectively. From September to November, the relative abundance of Chrysophyta and Chlorophyta increased, peaking on October 21. In the hepatopancreas samples from *M. mercenaria*, the relative abundance of Haptophyta was high on November 5 (34.55%). In the hepatopancreas samples from *M. meretrix*, the relative abundance of Euglenophyta was high on September 3 (15.79%). Only five phyla were identified in the hepatopancreas samples from *Ruditapes philippinarum*, with Cyanophyta being the most dominant group (> 99%). No representatives of the phyla Cryptophyta, Euglenophyta, Haptophyta, and Xanthophyta were detected in these samples (Fig. 4d).

**Figure 5:**
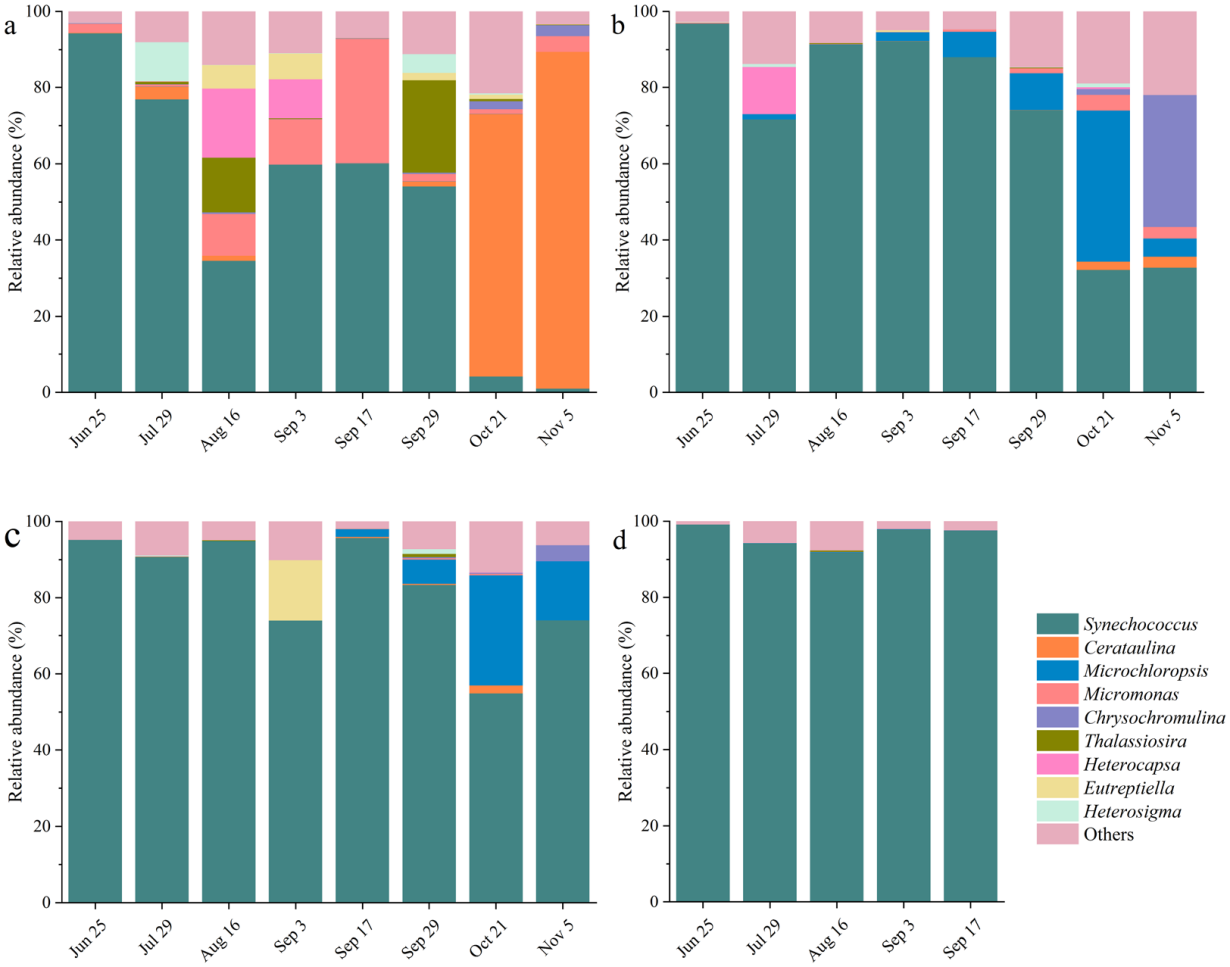
Temporal variation in the relative abundance of phytoplankton genera in the aquaculture water (**a**) and hepatopancreas samples of *M. mercenaria* (**b**), *M. meretrix* (**c**), and *R. philippinarum* (**d**) were identified with high-throughput sequencing.

A total of 78 phytoplankton genera were identified with high-throughput sequencing. In the aquaculture water, six dominant genera detected were *Synechococcus*, *Cerataulina*, *Micromonas*, *Thalassiosira*, *Heterocapsa,* and *Heterosigma* (Fig. 5a). *Synechococcus* was the most dominant genus from June to September, whose relative abundance ranged from 34.51% to 94.26%. On July 29, in addition to *Synechococcus*, *Heterosigma* was also a dominant genus. In August and September, the relative abundance of *Synechococcus* was less than that in June and July. The dominant genera also included *Micromonas*, *Thalassiosira,* and *Heterocapsa*. From October to November, *Cerataulina* became the most dominant genus, with a relative abundance that was over 68%. In the *M. mercenaria* hepatopancreas samples, the dominant genera detected included *Synechococcus*, *Microchloropsis*, *Chrysochromulina* and *Heterocapsa* (Fig. 5b). *Synechococcus* was the most dominant genus from June to September, which had a relative abundance ranging from 71.62% to 96.84%. On July 29, the dominant genus included *Heterocapsa* in addition to *Synechococcus*. From October to November, *Synechococcus* was still dominant, but their relative abundance had declined. In addition to the dominant genus *Synechococcus*, *Microchloropsis* was dominant on October 21 (39.66%), while *Chrysochromulina* was dominant on November 5 (34.55%). In the *M. meretrix* hepatopancreas samples, the three dominant genera included *Synechococcus*, *Microchloropsis* and *Eutreptiella* (Fig. 5c). *Synechococcus* was the most dominant genus throughout the experiment, with a relative abundance from 54.85% to 95.64%. On September 3, *Eutreptiella* was a dominant genus in addition to *Synechococcus*. From October to November, *Synechococcus* was still the dominant genus, but their dominance had declined. The relative abundance of *Microchloropsis* increased over 15%, indicating it was the second most dominant genus in the tested samples (Fig. 5c). In the *R. philippinarum* hepatopancreas samples, *Synechococcus* was the only dominant genus, accounting for more than 91% of the total phytoplankton abundance (Fig. 5d).

**Figure 6:**
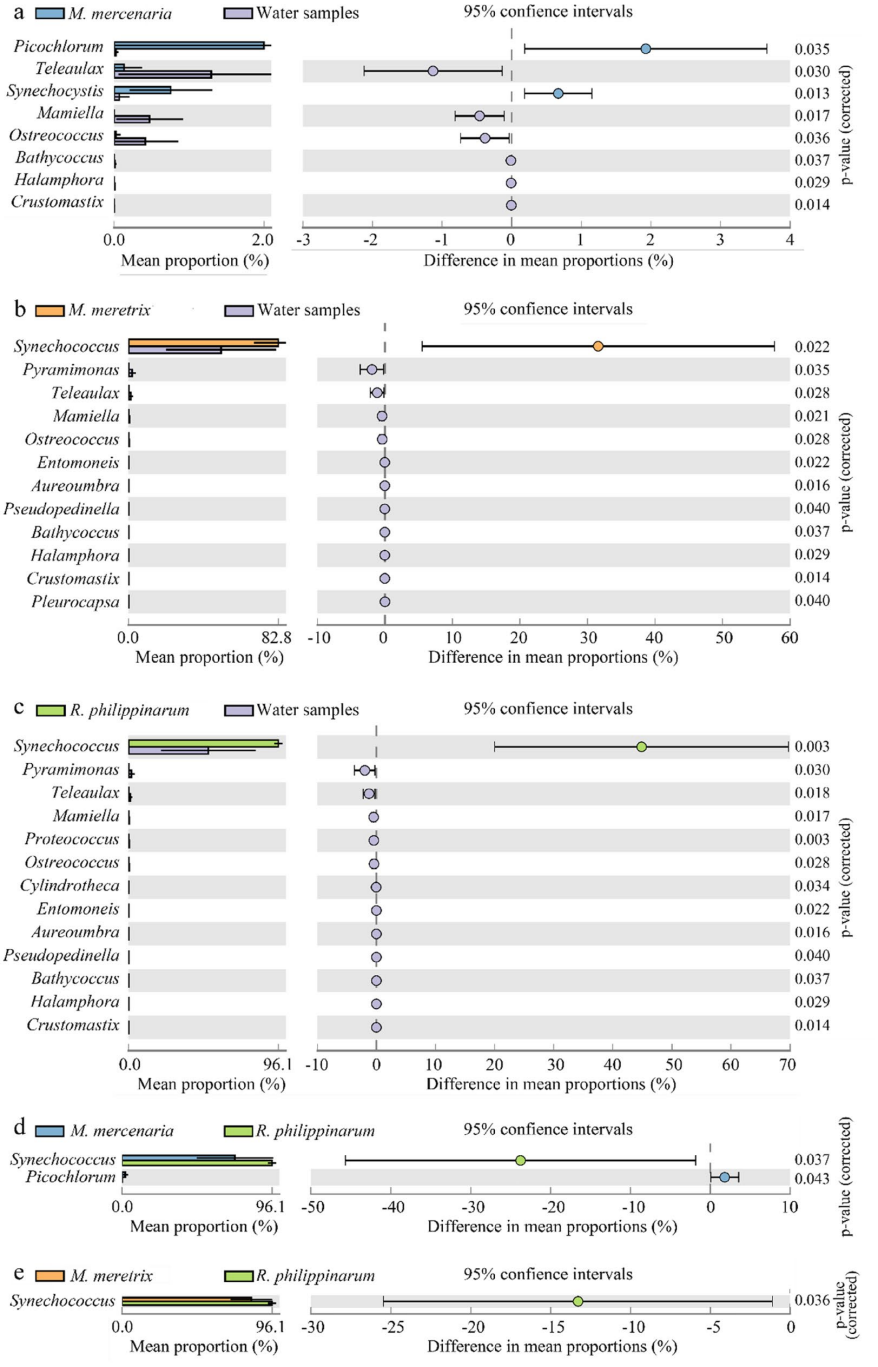
Extended error bar plots showing phytoplankton at the genus level that differed significantly between the *M. mercenaria* hepatopancreas and water samples (**a**), *M. meretrix* hepatopancreas and water samples (**b**), *R. philippinarum* hepatopancreas and water (**c**), between the hepatopancreas samples of *M. mercenaria* and *R. philippinarum* (**d**), and between the hepatopancreas samples of *M. meretrix* and *R. philippinarum* (**e**).

There were eight genera that differed significantly between the *M. mercenaria* hepatopancreas and water samples, including the five chlorophytes *Picochlorum*, *Mamiella*, *Ostreococcus*, *Bathycoccus* and *Crustomastix*, one diatom *Halamphora*; one cryptophyte *Teleaulax*; and one cyanophyte *Synechocystis* (Fig. 6a). There were 12 genera that differed significantly between the *M. meretrix* hepatopancreas and water samples, including five chlorophytes *Pyramimonas*, *Mamiella*, *Ostreococcus*, *Bathycoccus* and *Crustomastix*; two diatoms *Entomoneis* and *Halamphora*; two cyanophytes *Synechococcus* and *Pleurocapsa*; two chrysophytes *Aureoumbra* and *Pseudopedinella*; and one cryptophyte *Teleaulax* (Fig. 6b). There were 13 genera that differed significantly between the *R. philippinarum* hepatopancreas and water samples, including five chlorophytes *Pyramimonas*, *Mamiella*, *Ostreococcus*, *Bathycoccus* and *Crustomastix*; three diatoms *Cylindrotheca*, *Entomoneis* and *Halamphora*; two cryptophytes *Teleaula* and *Proteomonas*; two chrysophytes; *Aureoumbra* and *Pseudopedinella*; and one cyanophyte *Synechococcus* (Fig. 6c). There were no genera that differed significantly between the *M. mercenaria* and *M. meretrix* hepatopancreas samples (*p* > 0.05). There were two genera that differed significantly between the *M. mercenaria* and of *R. philippinarum*, hepatopancreas samples, including the cyanophyte *Synechococcus* and chlorophyte *Picochlorum* (Fig. 6d). Only the *Synechococcus* genus differed significantly between the *M. meretrix* and *R. philippinarum* hepatopancreas samples (Fig. 6e).

### Feeding selectivity in three bivalve species

**Figure 7:**
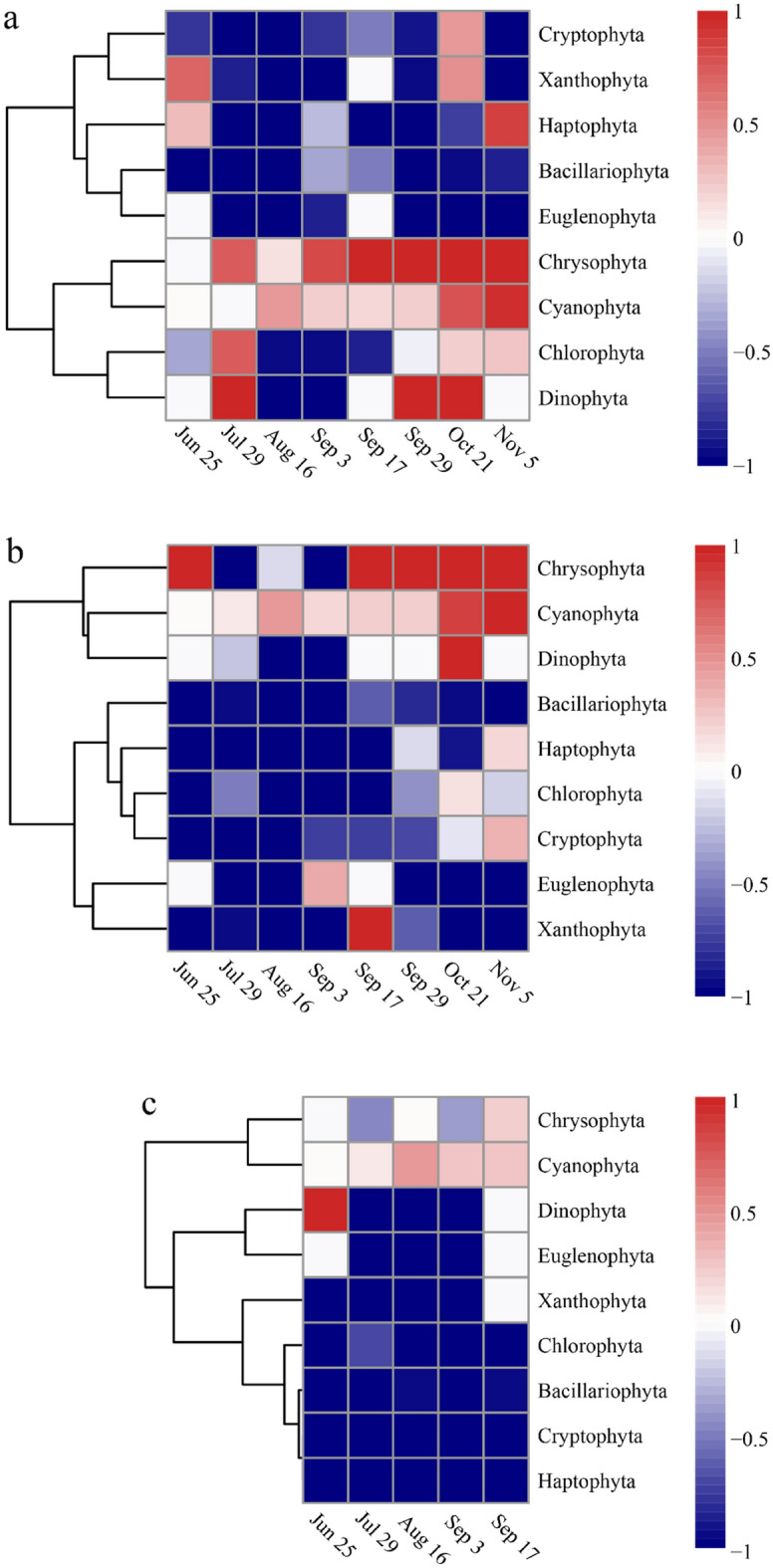
Heatmaps showing feeding selectivity of *M. mercenaria* (**a**), *M. meretrix* (**b**) and *R. philippinarum* (**c**) on phytoplankton at the phylum level.

The feeding selectivity of three bivalves on phytoplankton at the phylum level was evaluated using Ivlev’s electivity index (Fig. 7). The index values indicated that both *M. mercenaria* and *M. meretrix* showed preferential selection for Chrysophyta and Cyanophyta (Fig. 7a, b). Moreover, Chrysophyta and Cyanophyta increased with the growth and electivity index of *M. mercenaria* and *M. meretrix*. *M. mercenaria* avoided Bacillariophyta and Euglenophyta, while *M. meretrix* avoided Bacillariophyta, Haptophyta, and Chlorophyta throughout the aquaculture process. *Ruditapes philippinarum* preferentially selected Cyanophyta, but its electivity index was low. Additionally, *R. philippinarum* avoided Haptophyta, Cryptophyta, Bacillariophyta, Chlorophyta, Xanthophyta, and Euglenophyta throughout the aquaculture process (Fig. 7c). With respect to the number of phytoplankton groups selected, *M. mercenaria* selected the most, followed by *M. meretrix* and *R. philippinarum*.

**Figure 8:**
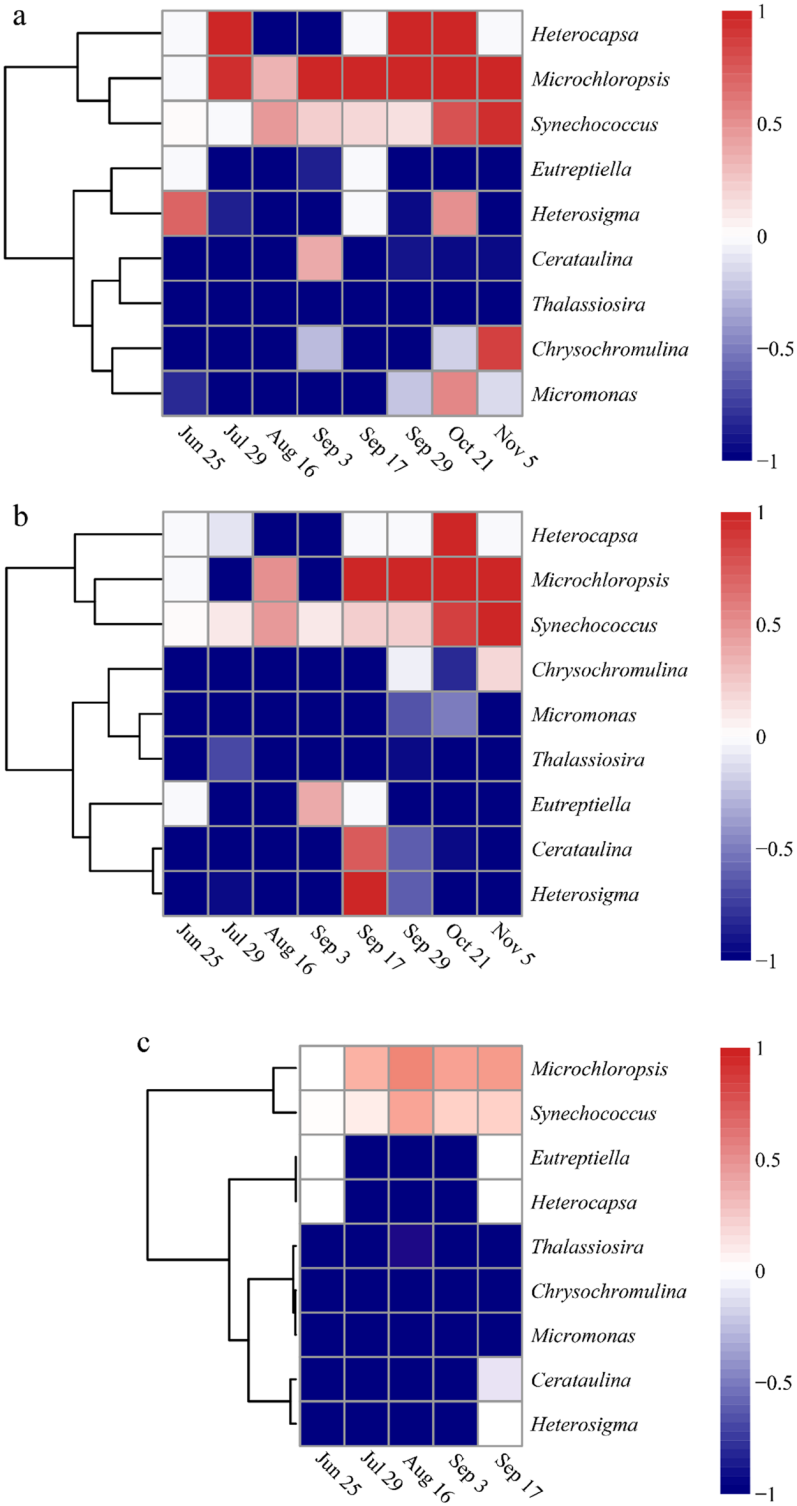
Heatmaps showing the feeding selectivity of *M. mercenaria* (**a**), *M. meretrix* (**b**), and *R. philippinarum* (**c**) on phytoplankton at the genus level.

**Figure 9:**
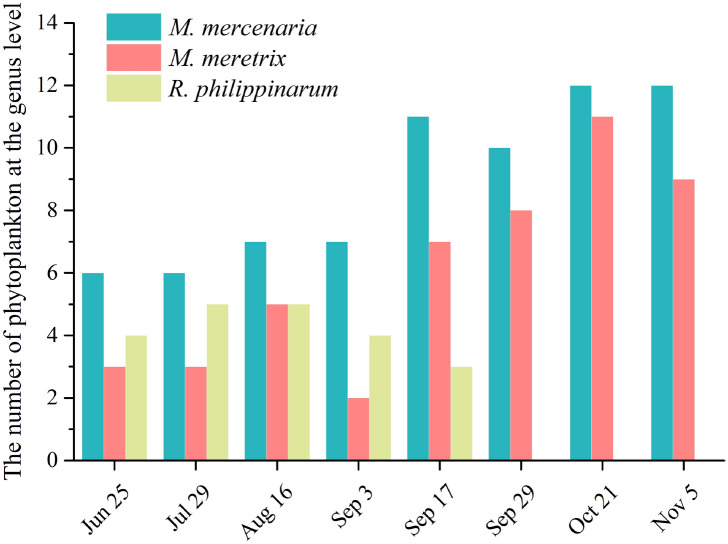
The number of phytoplankton at the genus level selected by *M. mercenaria*, *M. meretrix* and *R. philippinarum* grown in aquaculture.

At the genus level, *M. mercenaria* showed preferential selection for *Microchloropsis* and *Synechococcus* (Fig. 8a). The electivity index of *M. mercenaria* for *Microchloropsis* and *Synechococcus* increased with bivalve growth, while *M. mercenaria* tended to avoid *Thalassiosira* and *Eutreptiella*. Additionally, *M. meretrix* showed preferential selection for *Synechococcus* and its selectivity index increased with the growth of *M. meretrix* (Fig. 8b). At the later stage of the experiment, *M. meretrix* had high selectivity to *Microchloropsis*. We also found that *M. meretrix* avoided *Thalassiosira* and *Micromonas* throughout the aquaculture process (Fig. 8b), while *R. philippinarum* was found to preferentially select *Microchloropsis* and *Synechococcus* (Fig. 8c); however, its electivity index was low. *Ruditapes philippinarum* avoided *Thalassiosira*, *Chrysochromulina* and *Micromonas* throughout the aquaculture process (Fig. 8c). By comparing the number of selected phytoplankton at the genus level, we found that *M. mercenaria* had the most, followed by *M. meretrix* and *R. philippinarum*. With the growth of *M. mercenaria* and *M. meretrix*, more types of phytoplankton could be ingested (Fig. 9).

## Discussion

Earlier research found that larger particles could be captured more efficiently and removed from suspension faster by most bivalves when compared with smaller particles^9,38–40^, potentially because smaller particles were not efficiently retained by the gills of bivalves^21,41,42^. Conversely, other studies have reported that bivalves can capture smaller particles at a higher efficiency than larger particles^43^. The results from direct sampling methods indicated that mean capture efficiency of 2–4 μm particles ranged from 40 to 80% in mussels, and 20–60% in scallops^40,43,44^. Moreover, *M. mercenaria* was reported to clear chlorophyte and cyanobacterial picophytoplankton 1–4 μm in diameter from suspension with absorption efficiencies that ranged from 17.6 to 31.1%^45^. Therefore, the contribution of picophytoplankton and small nanophytoplankton as food resources for bivalves might be greater than previously reported. Currently, more studies have investigated the contributions of picophytoplankton and small nanophytoplankton (< 4 μm) to bivalve growth^43,46,47^.

In the present study, *Synechococcus* was detected as the dominant genus in the hepatopancreas samples obtained from three different bivalves (Fig. 5), with diameters of only 0.6–2.1 μm^48^. *Microchloropsis salina* dominated the hepatopancreas samples from *M. mercenaria* and *M. meretrix* in the later stage of this experiment (Fig. 5). A previous study of *Microchloropsis salina* ultrastructure showed that this species has length and width dimensions of 3–4 μm and 1.5–1.7 μm, respectively^49^. In the hepatopancreas samples from *M. mercenaria*, *Chrysochromulina parva* was the dominant species on November 5 (Fig. 5b), whose cell size was 3–7.5 μm × 2–4 μm^50^. This dominant species is a picophytoplankton that was too small to detect using morphological methods. However, they are one of the most abundant phytoplankton components in aquatic ecosystems^51^. Moreover, *Synechococcus* sp. was found to contribute 5–31% of the carbon necessary to sustain bivalve larval growth and respiration^52^. Some picophytoplankton species such as *Microchloropsis* sp. (formerly *Nannochloropsis* sp.) are rich in essential fatty acids necessary for bivalve growth and survival^53^. Consequently, the importance of these small phytoplankton in bivalve diets should be reassessed, especially for some genera of picophytoplankton. Particle selection, including the types of particles chosen for ingestion and the possible mechanisms mediating selection, has been studied extensively and reported in the literature^15,16^. However, no mechanism has been proposed for bivalves to date that would account for the capture of smaller particles in preference to larger sized particles. Recently, studies have reported that the interactions between the physicochemical properties of particles and the mucus covering the pallial organs most likely mediate bivalve food choice^54–56^. In particular, hydrophobicity is hypothesized to play a role in the capture of smaller particles^44,57^. Characterization of the surface properties of several planktonic bacteria in the SAR 11 Clade found that they can be more hydrophilic than most other bacteria and microalgal species^57–60^, which might be one reason why the photosynthetic bacteria *Synechococcus*, which is in the SAR 11 family, were captured preferentially by three bivalve species compared with other algae reported in this study (Fig. 8).

Studies have shown that different bivalve species have different feeding preferences^15,17,41^. Zhang et al.^23^ selected *Skeletonema costatum*, *Chlorella* spp., *Phaeodactylum tricornutum*, *Isochrysis galbana*, *Teraselmis* sp., *Platymonas subcordiformis*, and mixed algae to conduct a laboratory feeding experiment in juvenile *M. mercenaria*. The results showed that *P. subcordiformis* and *Teraselmis* sp. could be used as main baits for juvenile hard clam based on the survival and growth rates of the clams, as well as the degree of difficulty in bait cultivation. In laboratory feeding experiments conducted by Tang et al.^17^, *M. meretrix* was provided with five different species of marine microalgae (*I. galbana*, *Dunaliella* sp., *P. tricornutum*, *P. subcordiformis*, and *Pavlova viridis*), either alone and in various mixtures; they found that the bivalve larvae were characterized by selective feeding behavior, with a preference for *I. galbana*. Xiao et al.^61^ studied the filtration feeding of *R. philippinarum* juveniles on four species of algae and found that the feeding rate for *P. subcordiformis* was the highest, followed by *Chlorella saccharophila*, *I. galbana* and *Chaetoceros muelleri*. These feeding experiments of bivalves were conducted in the laboratory, which could not reveal their diet in situ. In the present study, we performed high-throughput sequencing of the 23S rRNA gene to assess the feeding selectivity of *M. mercenaria*, *M. meretrix* and *R. philippinarum* on phytoplankton in the natural environment. The results indicated that *M. mercenaria* showed preferential selection for *Microchloropsis* and *Synechococcus*, and tended to avoid *Thalassiosira* and *Eutreptiella* (Fig. 8a); *M. meretrix* showed preferential selection for *Synechococcus*, and avoided *Thalassiosira* and *Micromonas* (Fig. 8b); while *R. philippinarum* preferentially selected *Microchloropsis* and *Synechococcus*, and avoided *Thalassiosira*, *Chrysochromulina* and *Micromonas* (Fig. 8c). By comparing the number of selected phytoplankton at the genus level, *M. mercenaria* was the most selective of the bivalve species, followed by *M. meretrix* and *R. philippinarum*. Bivalve feeding selectivity on phytoplankton varied by species and was likely dependent on ctenidial architecture and latero-frontal cilia/cirri microstructure^15^. Following examination with transmission and scanning electron microscopy of the latero-frontal tracts on the bivalve gills, Nuculidae, Mytilidae Semelidae, Petricolidae, Veneridae and Pholadidae were found to have latero-frontal tracts consist of compound eu-latero-frontal cirri coupled with simple pro-latero-frontal cilia; while Anomiidae, Pectinidae, Limidae and Pinnidae had latero-frontal tracts that consisted of simple cilia only^62–64^. The latter seemed to be less efficient at entraining particles than former bivalves. Particles are captured by direct interception with the ctenidial filaments, but retention of particles is likely enhanced by mucus present on the frontal surfaces or ordinary filaments^16,65^. Therefore, beyond the differences in ctenidial architecture and laterofrontal cilia/cirri microstructure, the production and composition of the mucus that covers the ctenidia and labial palps varied between species and might be another one reason for why different species had different phytoplankton feeding selectivity.

Studies have shown that the demand for microalgae was different in bivalves at different growth stages^3,10^. In general, the larval stages required high quality algae, while post-larvae could feed on lower quality algae but remained sensitive to the proper biochemical composition^3^. However, the adults were able to feed on a greater variety of microalgae^10^. It is well documented in the literature that filtration rate increases with bivalve body size^66,67^. In this study, we found that with the growth of *M. mercenaria* and *M. meretrix*, the electivity index of these two bivalves for Chrysophyta and Cyanophyta also increased (Fig. 8). The kinds of phytoplankton they could ingest increased with the weight of *M. mercenaria* and *M. meretrix* (Figs. 2, 8, 9). We hypothesized that the filter feeding organs of juvenile bivalves were not fully developed and resulted in low filtration efficiency on algae in early stages of aquaculture. As the bivalves matured and increased in size, their particle ingestion rates increased^68^.

## Conclusions

Using high-throughput sequencing, we identified *Synechococcus*, *Microchloropsis,* and *Chrysochromulina* as the dominant picophytoplankton genera in the hepatopancreas of *Mercenaria mercenaria*, *Meretrix meretrix,* and *Ruditapes philippinarum*, indicating a requirement for reassessing the importance of these pico-sized algae to bivalve diets. The three bivalve species had different preferential selection of phytoplankton genera, indicating specific selection and avoidance of particular types of algae during their development in aquaculture. Therefore, oriented cultivation of the bait microalgae based on the preferences of different bivalve species might be an important way to improve the yield of bivalves in a marine pond.

## Materials and methods

### Experimental site and management

A five-month trial was performed at Rizhao Kaihang Aquatic Products Co., Ltd., Shandong Province in eastern China (35°19′8″N; 119°24′40″E) from June to November in 2019. Three bivalve species *Mercenaria mercenaria*, *Meretrix meretrix,* and *Ruditapes philippinarum* were cultured in the same pond with an area of 0.53 hectares. *M. mercenaria* were cultured on April 10, 2019 with a mean stocking size of 1.00 g and stocking amount of 160 kg. *M. meretrix* were cultured on May 23, 2019 with a mean stocking size of 16.67 g and stocking amount of 150 kg. *R. philippinarum* were cultured on April 3, 2019 with mean stocking size of 0.33 g and stocking amount of 56.5 kg. The water supplied for bivalve farming was from a polyculture pond through a recirculating pipeline system. In the polyculture pond, *Fenneropenaeus chinensis* were co-cultured with *Portunus trituberculatus*, while *M. mercenaria* and *Cynoglossus semilaevis* were cultured together. No bait was cast during the experiment. *R. philippinarum* were harvested in mid to late September; *M. mercenaria* and *M. meretrix* were harvested after the experiment was completed.

### Sample collection and preparation

Water temperature, salinity, dissolved oxygen (DO) and pH in the aquaculture pond were measured in situ using a Yellow Springs Instrument (YSI) handheld device (YSI Incorporated, USA). Two-liter water samples were collected and prefiltered through a sieve with a pore size of 200 μm to remove large suspended particles, microzooplankton, and other large cells. Then, 1 L of filtrate fixed with 5 mL Lugol’s solution for phytoplankton identification and counting; 500 mL filtrate was further filtered through a 0.22-μm Millipore membrane to collect the phytoplankton for DNA analysis. Biological samples of *M. mercenaria*, *M. meretrix,* and *R. philippinarum* were collected simultaneously and temporarily stored at 4 °C prior to dissection. To reduce the diet profile differences among individual bivalves, the hepatopancreases from 15–20 individuals were pooled as a single composite sample, homogenized with a pestle in 5 mL microtubes, and stored at -80 °C until they were used for DNA analysis.

### Morphological analysis of phytoplankton from water samples

Water samples (1 L) containing plankton were fixed with 5 mL Lugol’s solution. Each sample was subsequently concentrated to 50 mL in the laboratory, and then stored in the dark at 4 °C until analysis. Phytoplankton species were identified, and cell numbers were counted using a phytoplankton enumeration chamber under an inverted microscope (Olympus CKX41, Olympus Corporation, Tokyo, Japan)^37^.

### High-throughput sequencing of phytoplankton and bioinformatics analysis

Total genomic DNA was extracted from all samples using a FastDNA spin kit for soil (MP Biomedicals, OH, USA), following the manufacturer’s instructions. The quality of the DNA was verified by gel electrophoresis in 0.5% agarose gels, and DNA concentrations were determined using a NanoDrop spectrophotometer (NanoDrop Technologies, Wilmington, DE, USA). The primers p23SrV_f1 (5ʹ-GGA CAG AAA GAC CCT ATG AA-3ʹ) and p23SrV_r1 (5ʹ-TCA GCC TGT TAT CCC TAG AG-3ʹ) were used to amplify the 23S rDNA gene^31^. Purified PCR products were sequenced at Majorbio Bio-Pharm Technology Co. Ltd. (Shanghai, China) using the MiSeq platform (Illumina, San Diego, USA).

The 23S rDNA sequences were processed using the QIIME (version 1.91) software package. The original sequence data were sorted into valid reads after demultiplexing and quality filtering, and the high-quality sequences obtained were clustered into operational taxonomic units (OTUs) with an identity threshold of 97% using UPARSE (version 7.1 http://drive5.com/uparse/) with a novel “greedy” algorithm that performs simultaneous de novo*-*based chimera filtering and OTU clustering. The taxonomy of a representative sequence of each OTU was assigned using the Basic Local Alignment Search Tool (BLAST) in the NCBI database (http://www.ncbi.nlm.nih.gov). Following the exclusion of bacteria (all non-cyanobacteria) and unclassified sequences, phytoplankton sequences were selected for analysis of community composition based on taxonomic information^37^. The sequencing data used in this study were archived in the Sequence Read Archive (SRA) of the NCBI database under accession number SRP300559.

### Feeding selectivity analysis

The selectivity of bivalves with respect to feeding on phytoplankton was evaluated using Ivlev’s electivity index (E)^69^, which was calculated using the formula E = (r_*i*_ − p_*i*_) / (r_*i*_ + p_*i*_), where r_*i*_ is the relative abundance of phytoplankton in the bivalve hepatopancreas samples, and p_*i*_ is the relative abundance of similar items in the water sample. The calculated values of E ranged from −1.0 to + 1.0, with −1.0 ≤ E ≤ -0.3 indicating an avoidance or inaccessibility of a prey item, -−0.3 < E < 0.3 indicating a random selection from the environment, and 0.3 ≤ E ≤ 1.0 indicating active selection^70,71^.
